# Blood Shift During Cough: Negligible or Significant?

**DOI:** 10.3389/fphys.2018.00501

**Published:** 2018-05-28

**Authors:** Antonella LoMauro, Andrea Aliverti

**Affiliations:** Dipartimento di Elettronica, Informazione e Bioingegneria, Politecnico di Milano, Milan, Italy

**Keywords:** cardiopulmonary resuscitation, blood flow, physiology, cough, double body plethysmography

## Abstract

**Rationale:** It was reported how forceful rhythmic coughing can provide effective blood flow during ventricular fibrillation without direct chest compression. This mechanism of cough-assisted cardiopulmonary resuscitation constitutes a form of “cardiac massage” secondary to the intrathoracic and intra-abdominal pressure changes during cough. We have previously shown that significant blood shifts (BSs) occurs from the thorax to the extremities during expulsive maneuvers and that abdominal pressure controls the outflow of blood from the splanchnic vasculature. This mechanism was called abdominal circulatory pump. BS was quantified by using double body plethysmography (DBP), which combines total body plethysmography and opto-electronic plethysmography.

**Aim:** We hypothesized that coughing activates also the abdominal circulatory pump, being an additional mechanism that displaces a circulatory output sufficient to maintain consciousness in a patient with a non-beating heart.

**Methods and Results:** We studied seven healthy subjects (age: 28.6 ± 2.5 years) during series of voluntary coughs at three different operating volumes: after a spontaneous tidal volume, at total lung capacity (TLC) and at an intermediate volume. BS from the thorax to the extremities were measured by DBP during quiet breathing and during cough at each operating lung volume. BS during cough resulted significantly higher than during quiet breathing (*p* < 0.05). During the compressive phase, the blood outflow is around 200 ml, whereas during the expulsive phase BS increased (*p* < 0.05) with increasing operating volume, being almost 700 ml at TLC. At lower operating volume it is almost 400 ml.

**Conclusion:** Deep, vigorous coughing and the consequent fluctuations in intra-thoracic and intra-abdominal pressure activate both the thoracic and the abdominal pump mechanism. The former leads the low-resistance pulmonary veins to empty into the left heart. The latter can generate a circulatory output from the splanchnic region, which acts as a blood reservoir, to other body tissues. These findings might help to better understand the cardiopulmonary interactions during cough, particularly in patients with unstable cardiac function, and the mechanism by which coughing during unstable cardiac rhythms can maintain consciousness in human subjects.

## Introduction

The role of cough is to maintain airway clearance. Its efficacy depends on intrathoracic and intra-abdominal pressures developed. The pressure swings during cough may have important hemodynamic effects. In the literature, there are some case reports, clinical and animal studies reporting how forceful rhythmic coughing can provide effective blood flow during unstable cardiac rhythms (ventricular fibrillation, asystole, or heart block) without direct chest compression for up to 40 s (Criley et al., 1976b; Niemann et al., 1980, 1985; Bircher et al., 1982; Miller et al., 1989, 1994; Rieser, 1992; Lipton and Forstater, 1993; Mitton, 1993; Saba and David, 1996; Petelenz et al., 1998; Fritsch-Yelle et al., 1999; Girsky and Criley, 2006; Jafary, 2008; Keeble and Tymchak, 2008).

A *“thoracic pump”* is the suggested mechanism responsible for blood flowing during cough (Rudikoff et al., 1980; Niemann et al., 1981). This mechanism of cough-assisted cardiopulmonary resuscitation constitutes a form of “cardiac massage” caused by the swings of intrathoracic and intra-abdominal pressure during cough phases (Davis, 1983; Criley et al., 1984, 1986; Niemann et al., 1984; Schultz and Olivas, 1986; Cohen et al., 1989; Bergmann, 1992; Commerford and Lawrenson, 1992; Mauer et al., 2000). Concisely, while coughing the self-induced fluctuations of intrathoracic pressure compress the pulmonary vascular beds. Systemic blood flow is therefore pushed through the heart that acts as a passive conduit (Niemann et al., 1980; Criley et al., 1986; Cohen et al., 1989).

Oscillations of venous return can also result from the contraction of the diaphragm. When the diaphragm contracts, its dome moves caudally. This piston-like movement can generate appropriate swings in abdominal pressure (Miller et al., 2005) able to produce a circulatory output as great as resting cardiac output from the splanchnic region, that acts as a blood reservoir. The diaphragm, therefore, has also a circulatory role and this mechanism is known as “*abdominal circulatory pump*” (Aliverti et al., 2009). When the abdominal muscles contract simultaneously with the diaphragm, the output of the abdominal circulatory pump augments abruptly. During exercise (Uva et al., 2016) and expulsive maneuvers when abdominal pressure (P_AB_) reaches up to ∼100 cmH_2_O for 0.5–1 s (Aliverti et al., 2009, 2010) a significant amount of blood is displaced from the splanchnic vasculature to the extremities.

The expulsive maneuvre is somehow similar to the expulsive cough phase (ECP), the third and last phase of cough. Cough starts with an inspiratory cough phase (ICP) followed by a compressive cough phase (CCP) during which the glottis closes and the abdominal pressure suddenly increases secondary to a strong contraction of the expiratory muscles against it. The glottis reopens, ECP occurs and a forceful expiratory flow is generated [Roussos (1995/2016)].

We hypothesized that coughing activates also the *abdominal circulatory pump* that becomes an additional mechanism that contributes to generate a significant circulatory output.

To test this hypothesis, we have designed a pilot study to understand if the amount of blood shift during cough is significant and if and how it changes during the three phases of voluntary cough. Secondly, we tested if the double body plethysmography (DBP) technique (Aliverti et al., 2009), the only method able to quantify the amount of blood exchanged between the trunk and the extremities, can be used also during cough. Thirdly, because operating volume, i.e., the volume reached at the end of the inspiratory cough phase is known to significantly influence peak cough flow (PCF) but not the pressures generated (Smith et al., 2012), we have also investigated if operating volume may influence the cardiopulmonary interactions during cough.

## Materials and Methods

The double plethysmography method was previously tested and described in detail (Aliverti et al., 2009).

In synthesis, the variations of body (V_B_) and trunk (V_TR_) volume were measured simultaneously. Both measures are sensitive to gas compression and heating in the lungs. Because V_TR_ changes also in case of blood shifts (V_BS_) between the trunk and the extremities, V_BS_ can be therefore calculated as the difference between V_TR_ and V_B_. V_BS_ was considered positive when the blood moved from the trunk to the periphery, negative when flowing in the opposite direction.

V_B_ was obtained by the integration of the flow in and out a home-made, transparent, variable-flow whole body plethysmograph measured by a pneumotachometer mounted at the top of the box. This flow signal was anti-filtered in order to correct for the dynamics of the box itself.

V_TR_ was measured through opto-electronic plethysmography (OEP system BTS, Milan, Italy). OEP is based on a motion analysis system comprised of infrared video cameras that provides the 3D coordinates of 89 retro-reflective markers placed on the trunk of the subject according to anatomical points from clavicles to pubis. V_TR_ was calculated using Gauss’ theorem and a dedicated geometrical model applied to the 3D coordinates (Cala et al., 1996).

**FIGURE 1:**
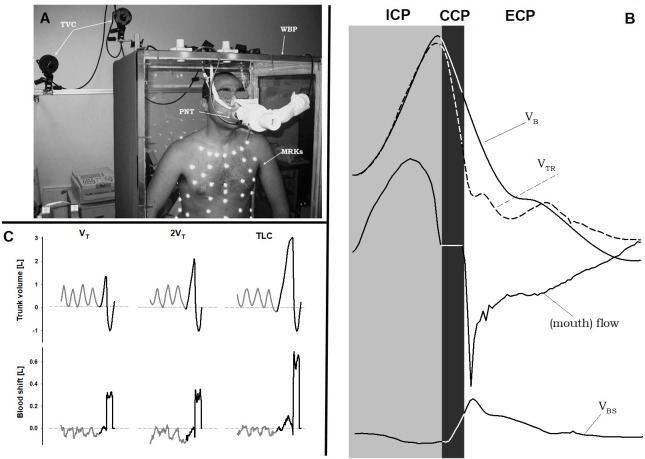
Double plethysmograph, traces and protocol. **(A)** Experimental set-up: the transparent whole body plethysmograph (WBP), the infra-red TV cameras (TVC), and the markers (MRKs) of optoelectronic plethysmography, and the pneumotacograph (PNT) to measure the flow at the mouth. **(B)** Time courses of body (V_B_) and trunk (V_TR_) volumes, flow measured at the mouth, and blood shift (V_BS_) during a single maximal voluntary cough. Gray area: inspiratory cough phase (ICP); black area: compressive cough phase (CCP); white area: expulsive cough phase (ECP). **(C)** Representative case of trunk volume changes (top panels) and blood shift (bottom panels) during spontaneous breathing (gray) and a single cough maneuver (black) at each operating lung volume: a tidal volume above functional residual capacity (V_T_, left panels), total lung capacity (TLC, right panels), and an intermediate volume (2V_T_, middle panels). V_BS_ was calculated as the difference between trunk and body volume variations. Positive values of V_BS_ indicate blood shifts occurring from the thorax to the extremities and vice versa. Written informed consent was obtained for the publication of the **(A)**.

Flow was also measured at the mouth using a linear heated pneumotachometer [3813 (0–800 L/min) Hans Rudolph, Inc., Shawnee, KS, United States] that connected the subject, sat inside the whole body plethysmograph, to the exterior of the box. This flow signal was used to distinguish the three cough phases (**Figure 1**): inspiratory cough phase, i.e., when the subject breathes in to insufflate the lungs (positive flow); compressive cough phase, i.e., when the flow is zero because of the closed glottis; and expiratory cough phase, i.e., when the flow becomes negative and PCF is reached.

Esophageal and gastric pressures were measured only in one subject by standard balloon-tipped catheters, connected to piezoresistive transducers (ASDX005D44D-A, full range scale ± 351 cmH_2_O, Sensortechnics Munich, Germany), respectively inserted in the esophagus and in the stomach (American Thoracic Society [ATS]/European Respiratory Society [ERS], 2002).

**Figure 1** shows the experimental set-up and representative tracings.

Seven healthy volunteers (age: 28.6 ± 2.5 years), two women, working in the laboratory and therefore having experience in respiratory maneuvers were studied.

After a period of spontaneous quiet breathing to familiarize with the instrumentation, subjects performed single maximal voluntary coughs every 40 s for three times. Coughs started from three different operating volumes (**Figure 1**) in a random order: after taking a spontaneous tidal volume above functional residual capacity (V_T_), at total lung capacity (TLC) and at an intermediate volume (2V_T_).

Time, V_BS_ and pressures were measured at the end of all the three cough phases.

The research protocol was approved by the local ethics committee of the INRCA Hospital, Casatenovo, LC, Italy and written informed consent was obtained from all the subjects who volunteered for the experiment.

To investigate the effects of the three operating volumes on flow, trunk volume, blood shifts and duration of compressive and expiratory cough phases, a one-way analysis of variance (ANOVA) was performed with operating volume as independent factor.

*Post hoc* tests were based on Holm-Sidak and Dunn’s method (SigmaStat 3.5, Systat Software, Inc., Richmond, CA, United States).

Significance was determined by *p* < 0.05.

## Results

The duration of inspiratory cough phase was similar (*p* = 0.29) between the operating volumes of V_T_ (1.03 ± 0.38 s) and 2V_T_ (1.59 ± 0.85 s), but it was significantly prolonged at TLC (2.94 ± 2.06 s, *p* < 0.01).

Similarly, when cough started from V_T_ and 2V_T_, the mean durations of compressive and expulsive cough phase were approximately 200 and 900 ms, respectively. On the other hand, when cough started at TLC, both phases significantly prolonged (CCP: 0.32 ± 0.18 s; ECP: 1.59 ± 0.70 s) and, accordingly, PCF occurred later.

**FIGURE 2:**
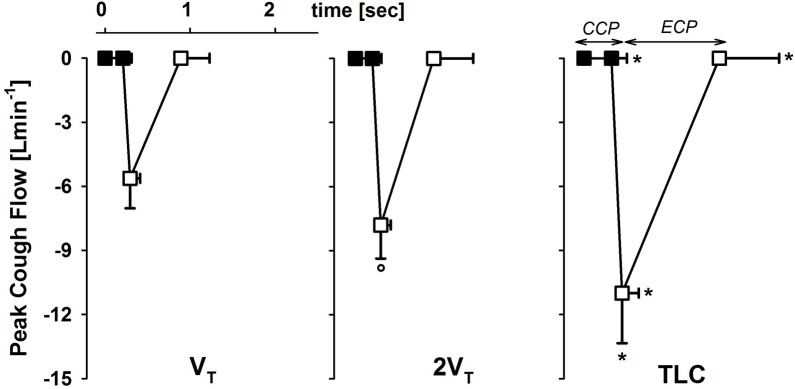
Average values ± standard deviation of peak cough flow at each operating lung volume: one tidal volume (V_T_) above functional residual capacity, left panel), total lung capacity (TLC, right panel), and an intermediate volume (2V_T_, middle panel). The x-axis displays the average values ± standard deviation of the duration of the compressive (CCP, close symbols) and the expulsive (ECP, open symbol) cough phases. ^∗^*p* < 0.05 vs. both V_T_ and 2V_T_; °*p* < 0.05 vs. V_T_.

PCF significantly increased with the operating volume. It almost doubled passing from V_T_ to TLC (**Figure 2**).

Although no visual feedback was provided, subjects reached three different operating volumes at the end of inspiratory cough phase. Accordingly, the compressive cough phase started from higher volumes with increasing operating volume. On the other hand, the lesser the operating volume, the lower chest wall volume at the end of the expulsive cough phase (ECP), indicating a stronger recruitment of the expiratory reserve volume.

**FIGURE 3:**
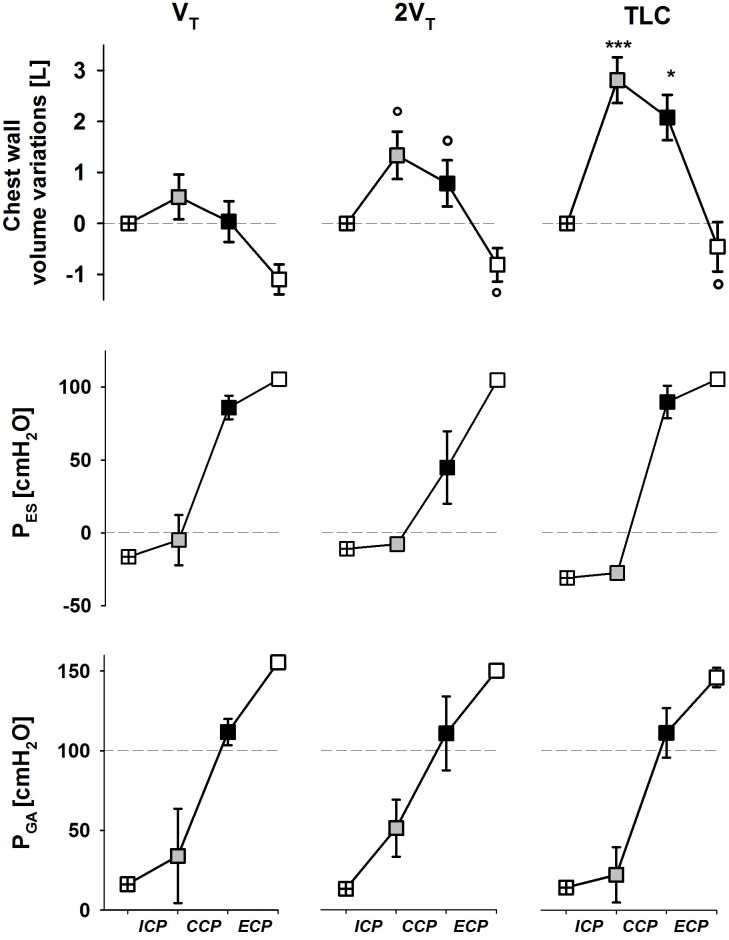
Average values ± standard deviation of chest wall (trunk) volume (upper panels), esophageal (middle panels) and gastric (bottom panels) pressures at start inspiration (crossed symbols), end of the ICP (gray symbols), end of the CCP (black symbols), and end of the ECP (white symbols) at each operating lung volume: a tidal volume above functional residual capacity (V_T_, left panels), total lung capacity (TLC, right panels), and an intermediate volume (2V_T_, middle panels). ICP, inspiratory cough phase; CCP, compressive cough phase; ECP, expulsive cough phase. ^∗^*p* < 0.05, ^∗∗∗^*p* < 0.001 vs. both V_T_ and 2V_T_; °*p* < 0.05 vs. V_T_.

Esophageal and gastric pressures did not significantly change in the three cough phases with operating volume (**Figure 3**).

During expiratory tidal breathing, blood shift was *circa* 51 ml, being significantly lower than the values found during compressive and expulsive cough phase. During CCP, V_BS_ was almost constantly around 200 ml independently on operating volume. On the other hand, during ECP the amount of blood propelled forward significantly increased with increasing operating volume reaching a mean value of about 700 ml at TLC.

**FIGURE 4:**
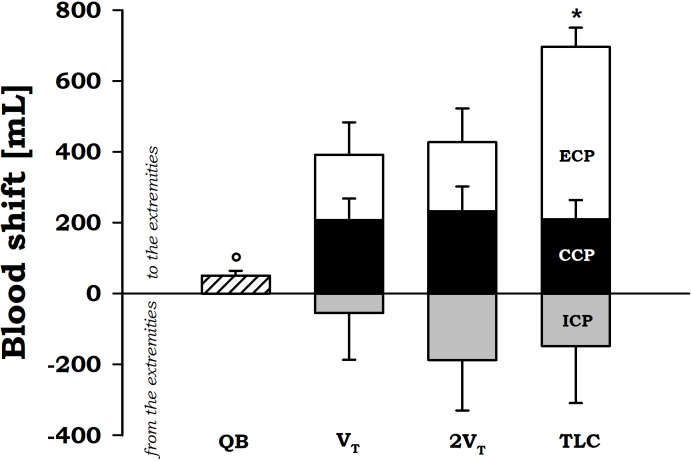
Average values ± standard error of blood shift during quiet breathing (QB) (measured during expiration), inspiratory cough phase (ICP, gray bars), compressive cough phase (CCP, black bars), and expiratory cough phase (ECP, white bars) at each operating lung volumes: a tidal volume above functional residual capacity (V_T_), total lung capacity (TLC), and an intermediate volume (2V_T_). ^∗^*p* < 0.05 vs. both V_T_ and 2V_T_; °*p* < 0.05 vs. both CCP and ECP.

No differences were found (*p* = 0.836) during inspiratory cough phase, presumably due to the high variability found (**Figure 4**).

## Discussion

The current pilot study is the first to quantify the blood exchanged between the trunk and the extremities during the different phases of single voluntary cough with increasing operating volume. We have demonstrated not only that blood shifts during cough can be measured, but also that their amount is considerable, therefore having potential important hemodynamic implications.

The main finding is that at the end of a single voluntary cough it is possible to force up to 700 ml of blood out the trunk toward the extremities. This happens only if the cough maneuvre starts from TLC. At lower operating volumes, the blood shift is about 400 ml that is still a considerable amount of blood available, among the others, for the heart and the brain.

These findings suggest that cough therefore has important effects on both the respiratory and the cardiovascular system. Cough not only is an important protective reflex, which helps to maintain airway clearance, but it also has important hemodynamic effects to be considered particularly in patients with unstable cardiac function. It ventilates the lungs, it maintains the patency and clearance of the airways and it produces systemic blood flow without cardiac compression.

The compressive phase of cough can be considered a short Valsalva maneuvers because expiratory muscles contract against the closed glottis (Boitano, 2006). Esophageal (intrathoracic) pressure reaches high positive values that compress the alveolar gas and displace 280 mL of blood out of the thorax independently on the operating volume.

During the expiratory cough phase, like during the expulsive maneuvres, abdominal pressure changes with a square-wave pattern. Because abdominal pressure is invariantly dependent on operating volume, we believe that time is the discriminant that makes V_BS_ reach 700 ml. When the operating volume is TLC, the duration of both the compressive and the expulsive phases increases. The former prolongs to 322 ms, the latter extends more than 1.5 s. The empting time constant of the whole splanchnic vascular bed is 610 ms (Aliverti et al., 2009). The compressive phase, therefore, is too short compared to it. On the other hand, during the expulsive phase the driving pressure has the time to empty the splanchnic reservoir and to displace augmented quantities of blood out of the thorax.

During the inspiratory cough phase V_BS_ is negative, therefore blood is pulled from the extremities to the thorax. When the diaphragm contracts, it makes pleural pressure become sub-atmospheric and abdominal pressure increase. The pressure gradient induced by the contraction of the diaphragm, therefore, has both a ventilatory (i.e., airflow into the lung to ventilate it) and a circulatory (i.e., an oscillatory composition of inferior vena caval blood) outcome. The hemodynamic effect of the diaphragm favors the splanchnic venous return during inspiration and the venous return of blood below the entry of the hepatic vein during expiration (Miller et al., 2005). The amount of blood shifted during the inspiratory cough phase was independent on the operating volume. The duration of the inspiratory phase was higher than the refilling time constant of the whole splanchnic vascular bed (570 ms, Aliverti et al., 2009) at all the considered operating volumes. Our results, therefore, suggest that during the inspiratory phase the splanchnic reservoir has enough time to refill completely.

One limitation of this pilot study is the absence of arterial pressure wave or cardiac output measurements, although compatible with the DBP, which could have helped understanding more in detail the hemodynamic effect of coughing on systemic circulation. Another limitation of the study is that esophageal and gastric pressures were measured only in one subject. All the considerations on the driving pressures can therefore result questionable, but the values found and the absence of correlation between peak pressures and operating volume are in line with the literature (Smith et al., 2012). For this reason, we think that these data provide an important message for the discussion and we decide to include them in this pilot study. Supported by the work of Smith et al. (2012), they suggest that blood shift seems independently variant on the driving pressure during cough. This further reinforces the role played by the time during the expiratory cough phase.

The primary aim of this pilot study was to verify the hypothesis that during cough a significant amount of blood is shifted from the thorax to the extremities, so contributing to a better understanding of the basic physiology of cough and its effects on circulation. For this reason, we have performed a pilot study on a small number of subjects deliberately chosen among those who were familiar with the experimental set-sup and able to control their operational volumes without any feedback. We have also verified the feasibility of the double body plethysmography technique to obtain these data.

The results of the present pilot study cannot be generalized, particularly to older people or to patients with cardio-respiratory pathologies, as only healthy young individuals with robust and strong cardiac and ventilatory pumps were studied. The authors believe that these preliminary results, however, help to better understand the physiological cardiac interactions of cough that ultimately allow a patient to remain conscious during unstable cardiac rhythms as reported in the literature.

Future studies, therefore, should be aimed to increase the population studied, not just in terms of number of subjects but also in its variety, particularly including elderly individuals, whose respiratory muscles may be weaker therefore generating comparably lower pressures, i.e., the forces for the blood movement. It would be interesting to extend this study not only to patients with impaired respiratory pump, but also to patients with cardiac pathologies, and therefore with altered cardiac pump, or both.

Because during cough the amount of shifted blood is far from being considered negligible, dedicated studies should be addressed to try to understand in which districts it flows, with particular interest to the brain and the heart. Nevertheless, our results suggest that deep and vigorous coughing and the consequent fluctuations in intrathoracic and intra-abdominal pressure not only activates the *thoracic pump mechanism* (Rudikoff et al., 1980; Niemann et al., 1981), leading the low-resistance pulmonary veins to empty into the left heart, but also the *abdominal pump* (Aliverti et al., 2009, 2010), displacing splanchnic blood to other body tissues. Moreover, the expulsive phase of cough not only compresses the heart to eject the blood, but also large vessels. We can speculate that this compression may be an additional factor shifting blood to the periphery. Our results provide the global amount of blood that is exchanged between the thorax and the extremities. Therefore, it takes into account also the blood squeezed by the large vessels, but it is not possible to distinguish it.

Although cough resuscitation is not the topic of the present pilot study and it is not intention of the authors to support its use in case of cardiac arrests or severe arrhythmias (American Heart Association, 2018), the results here reported seem to support the idea that vigorous coughing allows a patient to remain conscious during unstable cardiac rhythms giving time to the nurse or physician to intervene in settings such as the cardiac catheterization laboratory to end the dysrhythmia. Forceful cough, however, is a maximal volitional maneuvre that requires collaborative patient with efficient ventilatory pump, and that cannot be sustained for long period of time. The beneficial effect of cough-induced blood shift during a sudden arrhythmia, therefore, is not effective in all patients and for prolonged time.

During cough, the amount of blood shift is remarkable and therefore seems consistent with the hypothesis, provided by other authors, that hemodynamics during cough favors cerebral perfusion (Criley et al., 1976a,b; Niemann et al., 1980; Miller et al., 1989; Girsky and Criley, 2006). More studies, of course, are needed to investigate this aspect not to support cough-assisted cardiopulmonary resuscitation, but to better understand the hemodynamics during cough, that is a fundamental, natural protective reflex occurring in all individuals, including patients with cardiomyopathies.

Moreover, because usually cough occurs repetitively, future studied should focus on measuring V_BS_ during peals of cough. A first estimation of blood pushed out of the trunk during peals of voluntary coughing was published by Smith et al. (2012). They calculated V_BS_ by subtracting the volume of gas compressed in the lungs, estimated from Boyle’s law for isothermal transformations, and the volume expired at the mouth from the trunk volume provided by OEP. At the end of the first compressive phase, they found 350 ml of V_BS_ which rose to 613 ml at the end of the peal of cough. For this reason, we would expect blood shift even to increase because the presence of a competent aortic valve and peripheral vascular tone maintains a higher pressure in the aorta between coughs.

To summarize, cough is an important protective reflex, which helps to maintain airway clearance. It also has important hemodynamic effects to be considered, particularly in patients with unstable cardiac function. Deep, vigorous coughing and the associated fluctuations in intrathoracic and intra-abdominal pressure activate both the *thoracic* and the *abdominal pump* mechanism resulting in a significant amount of blood shift (about 200 ml during the compressive phase, and ranging from 400 to 700 ml during the expulsive phase).

## Author Contributions

AA conceived the original idea and designed the study. AL conducted the study, collected the data, performed the data analysis, interpreted the results, and drafted the manuscript. AL and AA approved the final version of the manuscript.

## Conflict of Interest Statement

The authors declare that the research was conducted in the absence of any commercial or financial relationships that could be construed as a potential conflict of interest.

## Abbreviations

CCP: compressive cough phase
ECP: expulsive cough phase
ICP: inspiratory cough phase
MRKs: markers
OEP: opto-electronic plethysmography
PCF: peak cough flow
PNT: pneumotacograph
TLC: total lung capacity
TVC: infra-red television (TV) cameras
V_BS_: blood shift volume
V_T_: tidal volume over functional residual capacity
WBP: whole body plethysmograph
V_B_: volume variations of body volume
V_TR_: volume variations of the trunk
